# Is a One Health Approach Utilized for Q Fever Control? A Comprehensive Literature Review

**DOI:** 10.3390/ijerph16050730

**Published:** 2019-02-28

**Authors:** Md Rezanur Rahaman, Adriana Milazzo, Helen Marshall, Peng Bi

**Affiliations:** 1 School of Public Health, The University of Adelaide, Adelaide, South Australia 5005, Australia; mdrezanur.rahaman@adelaide.edu.au (M.R.R.); adriana.milazzo@adelaide.edu.au (A.M.); 2 Adelaide Medical School and Robinson Research Institute, The University of Adelaide, Adelaide, South Australia 5005, Australia; helen.marshall@adelaide.edu.au

**Keywords:** Q fever, zoonotic disease, prevention and control, environmental, One Health, multi-sectoral

## Abstract

Q fever, a zoonotic disease transmitted from animals to humans, is a significant public health problem with a potential for outbreaks to occur. Q fever prevention strategies should incorporate human, animal, and environmental domains. A One Health approach, which engages cross-sectoral collaboration among multiple stakeholders, may be an appropriate framework and has the underlying principles to control Q fever holistically. To assess whether components of One Health for Q fever prevention and control have been applied, a comprehensive literature review was undertaken. We found 16 studies that had practiced or recommended a One Health approach. Seven emerging themes were identified: Human risk assessment, human and animal serology, integrated human–animal surveillance, vaccination for at-risk groups, environmental management, multi-sectoral collaboration, and education and training. Within the multi-sectoral theme, we identified five subthemes: Policy and practice guidelines, information sharing and intelligence exchange, risk communication, joint intervention, and evaluation. One Health practices varied between studies possibly due to differences in intercountry policy, practice, and feasibility. However, the key issue of the need for multi-sectoral collaboration was highlighted across most of the studies. Further research is warranted to explore the barriers and opportunities of adopting a One Health approach in Q fever prevention and control.

## 1. Introduction

Q fever, a zoonotic disease transmitted from animals to humans, is a significant public health problem worldwide. It is mostly occupationally acquired, and despite the availability of a vaccine for human use, at least in Australia, some countries continue to bear a substantial disease burden [1,2]. The annual incidence of Q fever notifications in the USA ranges from 0.28 to 2.40 cases per million persons. The reported incidence in England and Wales is similar to that in the USA. However, the annual reported incidence in Australia is higher with 15–49 cases per million persons [3]. 

The high incidence of infection in humans together with potential for spread through animal movements, magnitude of animal and human involvement, suboptimal national preparedness for outbreak control, and diagnostic challenges make Q fever control an important international public health priority [4,5]. Furthermore, infection in animals is associated with abortion storms particularly in goats, livestock culling, and reduced milk and meat production [6]. Reduced livestock production combined with human health costs derived from clinician visits, laboratory testing, hospital admission, and lost productivity signifies the impact of Q fever warranting an international response [6,7].

On average, an acute Q fever infection can cost a patient 7.5 days off work [6]. In an Australian study the cost of compensation claims from Q fever was estimated to be >A$3 million per annum, which today is approximated at A$4.3 million per annum, given inflation rates of the Australian dollar over 15 years [8]. Though immunization can largely abate these costs, screening of prior immunity through serology and skin tests, followed by vaccination if non-immune, is associated with high costs (≈A$300), and these costs are often responsible for lower immunization rates among at-risk occupational groups such as abattoir workers and farmers [9,10].

Considering the human-animal interface of zoonotic diseases, a One Health approach provides a strong framework in dealing with the economic challenges associated with Q fever [2,11,12,13]. One Health holistically engages human, animal, and environmental health professionals in collaborating nationally and globally for the pursuit of healthy living of humans and organisms [14]. Coordination and collaboration includes improving human surveillance, instituting animal surveillance and ensuring data sharing and intelligence exchange between veterinary and public health agencies, establishing communication, improving clinicians’ knowledge and attitude toward Q fever management, strengthening laboratory facilities, improving veterinary control measures, environmental monitoring, human and animal sero-surveillance, and access to screening and vaccination [11,15].

The aim of this review was to examine whether a One Health approach to Q fever control was applied and to identify gaps in practice and recommendations. One Health components that were considered for this review include human and animal serological surveys; knowledge, attitude, and practices among practitioners and farmers; One Health literature reviews; ecological correlations using multi-sectoral data; and outbreak investigations involving human, animal, and environmental domains.

## 2. Materials and Methods

### 2.1. Search Strategy

In order to identify all published studies on Q fever that utilized one or more components of a One Health approach, a systematic literature search was conducted in CINAHL, Embase, PsycINFO, PubMed, Scopus, and Web of Science databases until 13 June 2018. Searches were restricted to English language only. A logic grid using indexing languages (Emtree, MeSH) and/or keywords was developed for each database (see Table S1—Supplementary file for detailed search strategy). Keywords such as “Q fever” and “One Health”, their synonyms and closely associated words were used. Additionally, references cited in the included studies were pearled for possible relevance. Because a limited number of studies applied a One Health approach to Q fever, the literature search was extended to include conference abstracts and proceedings.

### 2.2. Eligibility Criteria

Studies that met one of the following two criteria were included:

- Studies that described the practice of one or more components of One Health in Q fever prevention and control;
- Studies that did not practice but recommended a One Health approach to Q fever prevention and control.

Excluded studies were those not having a One Health practice and/or recommendation focus in Q fever control. Books and book chapters were also excluded.

### 2.3. One Health Practice, Recommendation, and Observed and Expected Outcomes

Studies including serological surveys, outbreak investigations, ecological correlation, and systematic reviews that adopted a One Health approach from the outset were considered as practice. In contrast, published literature that recommended this approach for Q fever control was considered as recommendation. As highlighted in Table 1, One Health practices resulted in observed outcomes whereas recommendations were made with expected outcomes. 

## 3. Results

**Figure 1:**
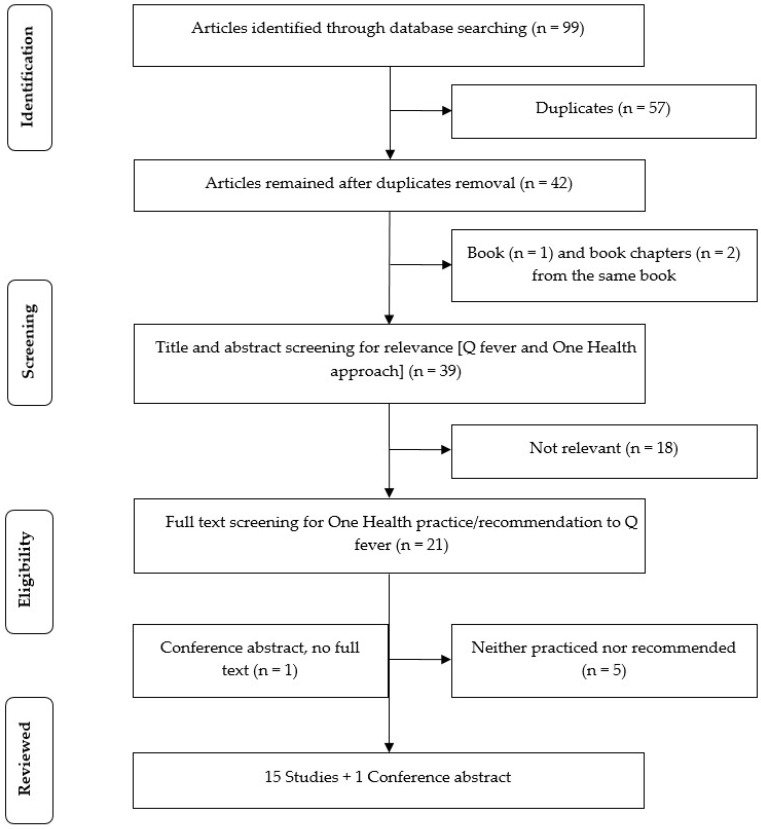
PRISMA flow diagram of the study selection process.

**Figure 2:**
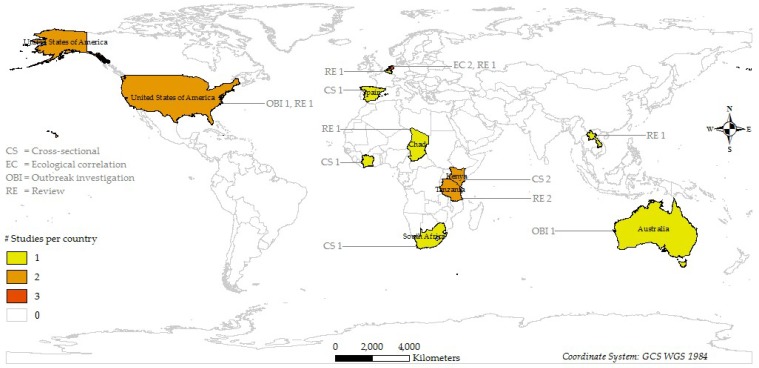
Distribution of studies that used a One Health approach to Q fever by location and study design.

Sixteen studies (15 full publications and 1 conference abstract) from 2009 to 2018 were included in this review. The earliest One Health study was published in 2009. A PRISMA flow diagram as shown in Figure 1 illustrates the study selection process. Four types of studies were included in this review: Cross-sectional study (*n* = 5), ecological study (*n* = 2), outbreak investigation (*n* = 2), and review (*n* = 7). Most studies were conducted in Africa (*n* = 7) and Europe (*n* = 5). While all cross-sectional studies were conducted in these regions, outbreak investigations were carried out in Australia (*n* = 1) and the USA (*n* = 1). Figure 2 shows the distribution and design of the studies. A summary of the studies including their location, study type, whether One Health approach was practiced and/or recommended, observed and/or expected outcomes, and comments on their strengths and weaknesses is given in Table 1.

The major themes elicited from this review were human disease risk, human and animal serology, integrated surveillance, vaccination, environmental management, multi-sectoral collaboration, and education and training.

### 3.1. Q Fever Risks to Humans

Human disease risks were examined by nine studies. Occupational risks included working in abattoirs; veterinary practices; farming, particularly goat farming; and transporting of infected livestock [17]. In the two Q fever outbreaks, livestock contact with manure and birth products was associated with human disease (RRs = 2.7 and 5.65) [24,29]. Additionally, in the USA, family members with frequent livestock contact (RR = 4.8) and in Australia those working in the office or close to the dairy without air filters (RR = 5.49) were found to be associated with Q fever. Proximity, defined as living within 1 kilometer of a farm with infected animals, was a risk factor in the Netherlands Q fever outbreak (RR = 46) [25]. These results suggest that occupational and environmental factors are pivotal in Q fever transmission.

### 3.2. Human and Animal Serology

#### 3.2.1. Human

Serological testing was carried out in seven studies. Of the seven studies, two performed human serology, one animal serology, and four both human and animal serology. In South Africa, 28/73 (38%) non-malarial febrile patients and 39/64 (61%) farmers, herders, and veterinary workers were *Coxiella burnetii* IgG positive [16]. In a Q fever outbreak in Australia, 32 (31%) individuals had unknown/no screening results. Of the remaining 72 cases with available results, 42 (58%) had positive Q fever serology [24]. In another outbreak in the USA, 81/135 (60%) persons had positive Q fever serology [29,30]. Contrary to the high seroprevalence among these occupational groups, the seroprevalence in a Kenyan community (*n* = 2049) was 2.5% [21].

#### 3.2.2. Animal

Animal serological studies found that 13.9% of cattle, 12.4% of goats, and 9.4% of sheep were *C. burnetii* seropositive in West Africa [18]. In Kenya, 10.5% of cattle, and 15% of goats in the Australian outbreak were seropositive [21,24]. A Spanish study found 22%–33% of European wildcats, Spanish ibex, and domestic sheep, and less than 2% of other species were seropositive [20]. These results underscore the importance of human and animal serology in quantifying Q fever risks and designing targeted control measures.

### 3.3. Integrated Q Fever Surveillance

**Figure 3:**
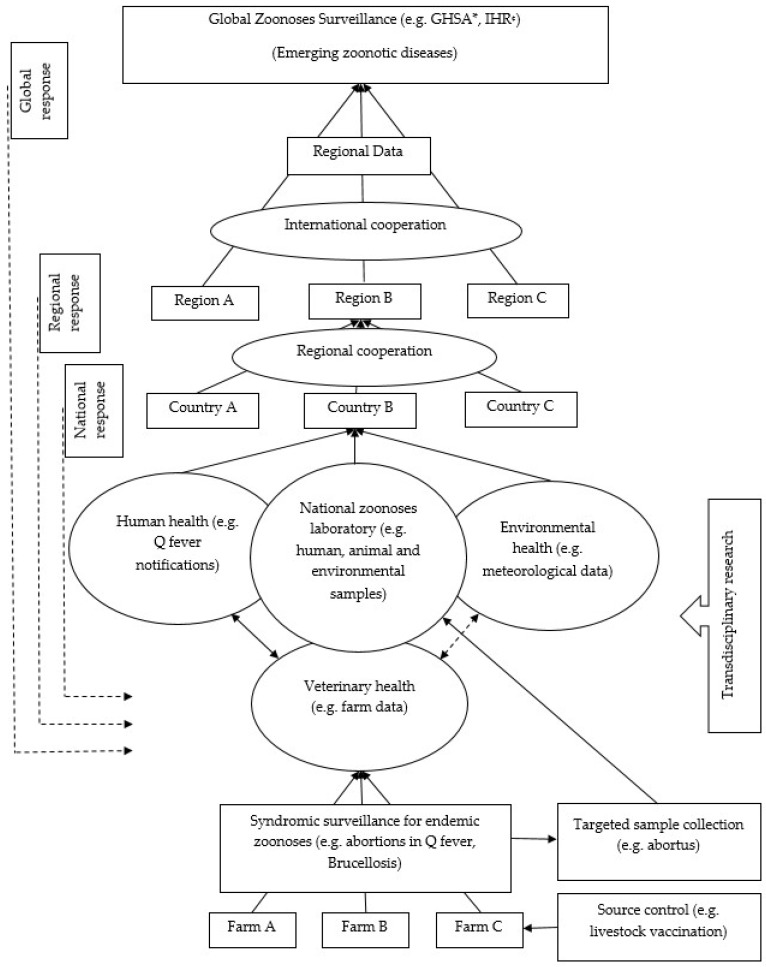
Schematic illustration of a One Health global zoonoses surveillance system. Conceptualized from [19,26,27,32]. * GHSA: Global Health Security Agenda; ^¢^ IHR: International Health Regulations.

Seven studies have shown that an integrated animal–human surveillance system by veterinary and public health authorities offers better disease monitoring than siloed surveillance systems [5,19,24,25,26,27,28,32,33]. Bond et al. [24] used integrated surveillance during their outbreak investigation in Australia and kept it under operation after the investigation was over. An integrated surveillance system can address multiple similar zoonoses simultaneously with the existing workforce. For example, appropriately trained farmers can use a syndromic approach such as animal abortions for considering Q fever, brucellosis, leptospirosis, and borreliosis and reporting this to veterinarians and human health authorities. This cost-effective surveillance system provides regional zoonotic data that can be used for global zoonotic disease surveillance priorities as shown in Figure 3 [19,26,27]. Integrated surveillance systems should have an integrated diagnostic facility where samples from a range of sources including human, animal, and environmental are tested guiding coordinated decision making and responses (see Figure 3) [32]. Unfortunately, an integrated Q fever surveillance system has rarely been implemented, except in a few circumstances such as in the San Diego County laboratory that has coordinated diagnostic facilities [32].

### 3.4. Vaccination

Vaccination was practiced and/or recommended in five studies, of which four recommended livestock vaccination. Human vaccination was extensive in the Australian outbreak and was effective in reducing human cases [24]. The authors recommended mandatory human vaccination for those having occupational contact with livestock. In contrast, livestock vaccination is a cost-effective intervention because it provides human health benefits through source control [19,31]. This can be carried out at farm levels or at livestock markets where *C. burnetii* contamination is high [19,21]. However, the available livestock vaccine is limited because of its biosecurity risks [24]. In the Australian outbreak investigation, these risks were considered and livestock were not vaccinated, as was the case in the Netherlands outbreak [24]. No study has shown the efficacy of livestock vaccination or the quantified associated biosecurity risks.

### 3.5. Environmental Management

Six studies practiced environmental management toward Q fever prevention and control including environmental sample testing (*n* = 2), environmental data analysis (*n* = 3), and an environmental risk factor review. Twenty eight (61%) of the 46 swab samples taken from the vagina and birth products of goats were *C. burnetii* positive in the Australian outbreak. However, air and bedding samples from the farm were not positive [24]. In the USA outbreak, 17%–26% of goat samples, 2%–7% of cattle samples, and the bulk tank milk filters were positive for *C. burnetii*. Though fecal samples were negative, 8/26 (31%) of the environmental samples including birth products, carcass, and manure were positive [30]. Environmental measures in the Australian outbreak investigation included manure storage in litter sheds, followed by composting and removal; immediate removal of aborted materials; and notifying goat buyers about the Q fever status of farms [24]. For an efficient Q fever control, an integrated surveillance system coupled with an environmental management component is warranted.

### 3.6. Multi-Sectoral Collaboration Including Joint Research 

Of the 16 studies, 13 (81%) directly discussed a multidisciplinary approach to Q fever control. Given the complex interactions between animals, humans, and the environment, a cross-disciplinary approach to Q fever control is required [17,26]. The results for this theme are categorized under the five subthemes discussed in the following sections.

#### 3.6.1. Policy and Practice Guideline Development

Nationally, Q fever control guidelines should be developed for health practitioners, industries, and their employees. For example, Simpson et al. [16] recommended an update of the conventional febrile treatment guidelines to include zoonoses such as Q fever. Countries also need to formulate specific agriculture- and husbandry practice-related policies at the national level [17]. While globally, the World Health Organization’s priority zoonotic diseases need to be revisited to include endemic zoonoses [27]. In terms of practice, guidelines and strategies to reduce human transmission were developed for patients, practitioners, and communities in the Australian and USA outbreaks [24,29]. Dunne and Gurfield [32] in their review showed how human and animal health laboratories were unified for testing a range of samples and coordinated decision-making. However, public health policies on Q fever control are limited except for those developed during outbreaks. 

#### 3.6.2. Information Sharing and Intelligence Exchange

Eleven (69%) studies discussed this subtheme. Knowledge of human, animal, and environmental domains provides opportunities for regular and planned interactions among stakeholders. This in turn builds trust, stewardship, and empowerment whereby disease control strategies are formulated through shared information and intelligence [16,17,19]. Moreover, such interaction opens the scope for transdisciplinary research that helps our understanding of the epidemiological and sociocultural complexities of Q fever [20,26,27]. For example, the Netherlands community Q fever outbreak in 2009 was also associated with a smaller outbreak in 2008. This recurrence was identified through the analyses of cross-disciplinary data [25]. Furthermore, sharing information and intelligence had demonstrated benefits in controlling both the Australian and USA outbreaks [24,29]. A joint diagnostic facility is, amongst others, a model par excellence because it offers greater access to information required for coordinated actions, as it is the functional endpoint of multiple related disciplines [32].

#### 3.6.3. Risk Communication

Five studies discussed risk communication. At the community level, risk information needs to be disseminated by both human- and animal-health authorities to increase the credibility of health messages. Credible messages may encourage individuals to refrain from risk behaviors such as sharing sleeping areas with livestock [16,23]. Likewise, risk communication through public–private partnerships reduces communication pitfalls and is cost effective [32]. In both the Australian and USA outbreaks, multidisciplinary risk assessment improved communication across stakeholders and helped formulate agreed risk reduction guidelines [24,29].

#### 3.6.4. Joint Intervention

Joint interventions, such as human and animal vaccination through cross-sectoral collaboration, provide superior disease control choices over a single approach [19,24]. These interventions are resource saving, devoid of duplication, and free from communication barriers [19]. In their outbreak investigation, Bond et al. [24] adopted this approach by including human vaccination, general biosecurity measures, and public health interventions.

#### 3.6.5. Evaluation

Periodic evaluation is crucial when a disease control program is implemented for possible adjustment of the program components [18]. However, program evaluations are not reported, and therefore studies are needed in future.

### 3.7. Education and Training Including Community Engagement

Six studies discussed this theme: Practitioners’ education and training (*n* = 2), community education and engagement (*n* = 3), and both (*n* = 1). Q fever knowledge was very limited among healthcare providers in Kenya. Most of them had no or poor knowledge about the disease, its transmission and treatment [23]. Medical and veterinary practitioners need updated knowledge about Q fever, risks of transmission, diagnosis, and management to educate their clients on how to prevent zoonotic diseases [16,30]. Likewise, community members, particularly at-risk populations, should be targeted for audiovisual educational promotion on how to reduce their zoonotic risks [18]. Educating the community is an integral part of zoonosis control as it provides individuals with informed choices for practicing risk reduction strategies. Additionally, this offers a socially purchased benefit of community trust and engagement [19,23]. If education providers are trustworthy, target groups take ownership of the zoonosis prevention process. An example is the educational campaigns for workers’ families in the Australian outbreak response whereby general practitioners were requested to promote optional vaccination among them [24].

## 4. Discussion

This review summarizes contemporary published evidence on using a One Health approach for Q fever prevention and control. Although Q fever is ubiquitously distributed [1,34], the contexts, magnitudes, and risks are not homogeneous. Therefore, One Health components and practices varied between studies. For example, the origin of the outbreak and delayed institution of an investigation were similar in the Australian and the Netherlands outbreak. However, Netherlands’ investigation was bigger in magnitude, culled animals, restricted ruminant breeding, and made animal notification mandatory [24,35]. Although an outbreak investigation per se may be less appropriate to generalize, all practices in this review contribute to a strong generic One Health model for Q fever prevention and control.

Despite the fact that Q fever infection may occur without occupational exposure, such as sporadic cases living in proximity to infected animals, our review has identified common occupational groups at risk including farmers, abattoir workers, and veterinarians [17,36]. However, apart from Bond et al. [24] no other studies acknowledged the occupational risks and advocated for mandatory vaccination of occupational contacts, most likely because the vaccine is only registered for use in Australia. Furthermore, the Australian investigation also addressed the environmental transmission through promoting vaccination among people living in the vicinity [24]. These findings emphasize that the extrapolation of vaccination practices is required to avoid further outbreaks.

Human vaccination is 97%–100% efficacious when given outside the natural incubation period [37,38,39]. However, high screening and vaccination costs and access to general practitioners are often viewed as challenges [9,40]. Some studies have shown that unlike human vaccination, animal vaccination is cost effective as it reduces shedding of the bacterium in animals, environmental contamination, and the likelihood of disease transmission to humans [19,31]. From a One Health perspective, concurrent human–animal vaccination at livestock markets would offer one of the best Q fever prevention strategies. It reduces *C. burnetii* contamination in animals and allows mass vaccination of farmers who perceive cost and access to care as barriers [9,19,21]. However, given that the available livestock vaccine has manufacturing biosecurity concerns, caution must be exercised in the event a concurrent vaccination program at livestock markets is planned.

Human serology plays an important role in quantifying Q fever burden. High seroprevalence among occupational groups in this review is similar to that of goat farmers in the Netherlands [41] and may indicate that Q fever prevention should target occupational contacts. Unlike this, low population seroprevalence is consistent with the Netherlands and USA national rates that makes the general population a less appropriate target for interventions [21,30,41]. In contrast, as animals are asymptomatic carriers [42], their serology can identify species that have previously been infected and can have some role in identifying flocks or herds where *C. burnetii* is endemic. However, it has been shown that there is no association between antibody response and shedding of the organism [18], which represents the true public health risk.

Given that Q fever is under-diagnosed and underreported, human surveillance is the most reliable option for burden estimation [43,44]. Animal surveillance is important because human outbreaks are preceded by animal infections that may manifest with abortions, warning public health professionals to activate an alert mechanism [33,34,45]. The integration of the two surveillance systems could reduce communication pitfalls, save resources, and provide zoonotic data for national and global coordination [19,26,27,32]. Although in the Netherlands an integrated surveillance system was instituted, it was challenged by inadequate coordination and lack of trust and stewardship between stakeholders [31]. Enserink [31] therefore argued that for the functionality of an integrated surveillance system stakeholders need to resolve all possible inter-sectoral disputes beforehand.

Another major domain of One Health is the environment that allows host–reservoir interactions, propagates disease transmission, and deserves meticulous consideration in Q fever control [17]. The fact that soon after shedding *C. burnetii* settles in dust, becomes aerosolized, and infects humans makes environmental management a key factor in disease control [7,46]. Such management practices varied between settings. For example, the Australian and the Netherlands outbreaks practiced manure management while the latter restricted humans and transports [24,35]. These measures were key to the successful control of both outbreaks [24,35] and, therefore, deserve inclusion in Q fever prevention and control practices.

Multi-sectoral collaboration is the central theme of this review. Although a majority of studies explicitly emphasized a multi-sectoral and collaborative approach, very few outbreak responses applied this in practice [11,32]. In the USA and Australian outbreaks, both countries lacked prior policies for collaboration. One reason is the enduring bureaucracies and disputes between veterinary, public health, and environmental sectors that hinder countries formulating and implementing the multi-sectoral policies identified by Enserink [31] in the Netherlands outbreak. This disintegration needs to be resolved ahead of time whereby heterogeneous stakeholders cooperate and collaborate on a homogenous platform. In reality, many countries are yet to have intellect and skill sharing that provides cross-sectoral data, ensures continued vigilance, and expedites timely response should an event surge [32].

Several studies have identified that inter-sectoral collaboration is the building block of joint risk communication. If risk communication to the community is conducted by different authorities individually, it is likely to confuse the community [19]. On the contrary, when joint risk communication is carried out, individuals feel that authorities are trustworthy and self-motivate themselves to follow health messages [16]. Moreover, joint risk communication could be a milestone for reforming a fragile health system [19]. It mediates the success of joint interventions by assisting individuals in making informed decisions. An example is the joint vaccination in Chad for mobile farmers’ children and their livestock. This intervention was cost effective and more importantly set a milestone for veterinary and public health coordination [28]. However, joint intervention is not limited to joint vaccination only as is observed in the Australian outbreak investigation where human vaccination was coupled with several public health actions [24].

Considering the complexities of practice where One Health programs are used, evaluation becomes mandatory for accommodating changes deemed necessary as the evidence evolves [47]. However, our review did not identify any such program evaluation. Finally, education and training of health practitioners and at-risk groups are crucial in shaping their attitude and practice related to Q fever prevention. Practitioners’ knowledge makes them vigilant as a high level of suspicion is required for Q fever diagnosis, given its inapparent clinical course [35,48,49]. Similarly, at-risk populations’ knowledge helps them refrain from practicing high-risk behaviors [8,19,23,50].

## 5. Conclusions

This review presents an up-to-date evidence base for controlling Q fever in a One Health approach. One Health programs need to be based on human, animal, and environmental domains. These programs are highly context specific and their success depends on their flexibility to incorporate required changes. Emerging themes may be employed alone or in a combination of different One Health programs based on intercountry policy, practice, and feasibility. However, as long as the holistic underpinning of the multi-sectoral collaboration is preserved, programs are likely to function well. Further research into the barriers and opportunities of adopting a One Health approach to Q fever prevention and control is warranted.

## Figures and Tables

**Table 1:** Characteristics of the studies that used one or more components of One Health in Q fever prevention and control.

Study and Location	Study Type	One Health	Observed and/or Expected Outcomes	Comments
[16] South Africa	Cross-sectional	**Practiced**		
		▪ Risk factor survey among farmers, herders and veterinary staff	▪ Q fever included in the differential diagnosis of febrile illnesses	▪ Diagnostic challenges related to febrile illnesses identified
		▪ Human serology	▪ Positive Q fever serology demonstrated	▪ Small sample size and non-random selection of participants limit generalizability of the results
		**Recommended**		
		▪ Education and training related to zoonosis for human health and veterinary practitioners	▪ Educated clients for better disease prevention	
[17] Europe/Belgium	Systematic review	**Practiced**		
		▪ Risk factors reviewed: ▪ Occupational factors e.g. farmers, abattoir workers ▪ Husbandry factors e.g. goat farming ▪ Environmental factors e.g. infected livestock transportation	▪ One Health is a model for Q fever control addressing complex interactions between the reviewed factors	▪ One Health focus was drawn from the Netherlands experience, which may fail to appreciate the subtleties of Q fever epidemiology that determine possible control options in other countries
		**Recommended**		
		▪ Q fever monitoring in high incidence countries	▪ Promote optimum health of humans, animals and environment	
		▪ Collaboration across disciplines		
[18] Côte d’Ivoire	Cross-sectional	**Practiced**		
		▪ Risk factor survey in rural farming communities	▪ Positive Q fever serology at the farm and community level	▪ No association between animal abortions and Q fever seropositivity contradicting findings in other studies e.g. Netherlands’ outbreak
		▪ Human and animal serology		
		**Recommended**		
		▪ Educate community about zoonosis by combining public health and animal health	▪ Reduced human exposures to Q fever	
[19] Africa/Tanzania	Review	**Recommended**		
		▪ Global zoonosis surveillance system	▪ Impromptu response to endemic zoonosis	▪ Stakeholders meet, interact, share experiences and embark on agreed upon decisions
		▪ Strengthen national core capacities	▪ Coordinated response to future disease threats	
		▪ Interventions targeted at Q fever source e.g. livestock vaccination	▪ Reduction of animal abortions and human Q fever cases	
		▪ Community trust, engagement and collaboration	▪ Less fragmentation, less inequalities for sustainable development	
[20] Spain	Cross-sectional	**Practiced**		
		▪ Wild and domestic ruminant serology	▪ Positive *C. burnetii* antibodies in wild and domestic ruminants ▪ First evidence of antibodies in European wildcats	▪ Inclusion of human serology would have provided a strong One Health practice and helped further understanding of Q fever epidemiology in Spain
		**Recommended**		
		▪ Multidisciplinary studies required	▪ *C. burnetii* epidemiology at human-livestock-wildlife interface will be better understood	
[21] Kenya	Cross-sectional	**Practiced**		
		▪ Risk factor survey among randomly selected households	▪ *C. burnetii* exposure was heterogeneous	▪ Studying only cattle limits extrapolation of results to settings such as the Netherlands where small ruminants are the main reservoir ▪ Without full explanation of socio-cultural factors, it is premature to conclude certain ethnic groups had increased exposure risks
		▪ Human and cattle serology	▪ Cattle brought from livestock markets had highest seroprevalence	
		▪ Spatial correlation of cattle and human seropositive samples	▪ Human and cattle seroprevalence was not associated	
		**Recommended**		
		▪ Livestock markets be targeted for Q fever control interventions (e.g. animal serology and vaccination)	▪ Reduction of *C. burnetii* shedding in previously exposed animals	
[22] Netherlands	Ecological correlation	**Practiced**		
		▪ Netherlands’ outbreak analyzed Q fever notification data, farm data and climate data	▪ Q fever notification was correlated with environmental conditions, e.g. wind current and humidity	▪ An estimated 8% of Q fever cases was notified in 2009 outbreak. This, in part limited the authors' conclusion of the causal associations between human notifications and environmental predictors
		**Recommended**		
		▪ Ecological research on outbreak associated data	▪ Spatially planned farming	
[23] Kenya	Cross-sectional	**Practiced**		
		▪ Knowledge, attitude and practices survey among medical, veterinary and wildlife workers, and farmers	▪ Q fever knowledge was low among most participants (94% human health providers had little or no knowledge)	▪ How stakeholders’ knowledge contributes to a One Health collaboration, and why this multi-sectoral approach is important is not discussed
		**Recommended**		
		▪ Provide healthcare professionals updated Q fever knowledge	▪ Effective control of Q fever	
		▪ Strengthen multi-sectoral collaboration		
		▪ Community sensitization	▪ Help community members prevent Q fever	
[24] Australia	Outbreak investigation	**Practiced**		
		▪ Multidisciplinary epidemiological investigation and animal serology	▪ Comprehensive risk assessment techniques and consensus control measures developed ▪ Workers protected by HEPA* filters ▪ Goats identified as likely source of the outbreak ▪ Controlled human cases without source control	▪ Key similarities with the Dutch outbreak include outbreak source, both occurred at goat farm; use of human vaccination; and application of a One Health approach. Differences include magnitude of the outbreaks, livestock vaccination was not used in the Australian outbreak because of manufacturing biosecurity concerns
		▪ Skin and serological testing for workers, subsequent vaccination	▪ Could not prevent infections in workers’ family members	
		▪ PCR testing of aborted materials, vaginal swabs, environmental samples	▪ Ongoing farm environmental contamination due to intensive breeding and milking goats demonstrated	
		▪ General measures e.g. biohazard sign erection	▪ Presumably these public health measures controlled the outbreak	
		▪ Site surveillance launched		
		▪ Health education		
		▪ Management of farm environment e.g. manure management		
		**Recommended**		
		▪ Mandatory vaccination for all occupational contacts	▪ Prevent acute Q fever cases	
		▪ Further research to identify possible interstate introduction of Q fever	▪ Traditionally held views that interstate importation of *C. burnetii* to Victoria may be established	
		▪ Validation of IFA	▪ Livestock and wildlife prevalence of *C. burnetii* could be established	
		▪ Livestock vaccination	▪ Reduced environmental shedding	
[25] Netherlands	Ecological correlation	**Practiced**		
		▪ Q fever notification data, veterinary and farm data analyzed	▪ Largest goat farm had abortion waves, bulk tank milk and almost all samples positive for *C. burnetii* – considered as the most likely source	▪ Largest goat farm caused a smaller outbreak in 2008, with a larger community outbreak following year ▪ Public health and veterinary health professionals should work together on an alert mechanism to identify any potential human Q fever outbreaks ahead of time
		▪ Largest farm visited, and farmers interviewed on risk factors	▪ Several unsafe farm practices related to manure and removal of birth products	
		▪ Atmospheric dispersion model used	▪ Likely period of infection and airborne propagation shown	
		**Recommended**		
		▪ Consider farms with history of *C. burnetii* infection as potential source of human outbreaks	▪ These could guide future Q fever control strategies	
		▪ Use meteorological forecast data		
[26] Africa/Tanzania	Feature/Review	**Recommended**		
		▪ Syndromic surveillance and targeted collection of diagnostic materials e.g. aborted products	▪ Better linking etiology and epidemiology of *C. burnetii* in humans and animals ▪ Early detection of possible human outbreaks ▪ Identification of key intervention points ▪ Cost-effective interventions	▪ One Health approach provides a holistic management perspective in a cost-effective fashion and is most viable option to minimize misdiagnosis, assess zoonotic impacts and utilize disease control methods
		▪ Improved communication across sectors	▪ Early diagnosis, prompt treatment and better control strategies	
		▪ Regional data on Q fever burden is essential	▪ Q fever becomes a global disease control priority	
[27] Lao People’s Democratic Republic (Laos)	Review	**Practiced**		
		▪ Summarized 8 pig associated zoonoses, their risks and impacts	▪ Misdiagnosis and underreporting were common	▪ Focusing only on pigs led the scope of wide range of zoonotic reservoirs remained unexplored. Inclusion of a range of reservoirs could have offered a stronger case scenario of advocating for a One Health approach ▪ Unique aspect is emphasizing socio-cultural determinants of zoonoses
		**Recommended**		
		▪ Improved diagnostic approaches	▪ Reduced diagnostic errors and improved notification	
		▪ Strengthen disease surveillance systems		
		▪ Interdisciplinary collaboration and research	▪ Designing socially and culturally appropriate control methods	
[28] Africa/Chad	Conference proceedings/Review	**Practiced**		
		▪ Summarized “One Health” studies among mobile farmers ▪ Linked human and animal health studies ▪ Summarized human and animal intervention (e.g. vaccination) studies ▪ Combined human and animal serological studies	▪ Livestock vaccination coverage higher than human vaccination in farming communities ▪ Better access to care for mobile farmers and their families ▪ Camel breeding associated with human *C. burnetii* seropositivity	▪ One Health programs were shown to be efficient (e.g. joint vaccination) and acceptable (e.g. health assessment using mobile phone). Public health and veterinary interventions which are coordinated, accessible, resource saving and based on community needs are successful
		**Recommended**		
		▪ Integrated zoonotic surveillance using cell phone for mobile farmers to be established	▪ Demographic and disease surveillance and control methods for mobile populations	
		▪ Social and anthropological studies	▪ Social and cultural complexities of zoonotic infections will be understood	
[29] USA	Outbreak investigation	**Practiced**		
		▪ Multidisciplinary outbreak investigation by veterinarians, public health nurses, medical doctors, epidemiologists and Q fever and reference diagnostic laboratories	▪ Extent and epidemiology of this outbreak was determined	▪ A good example of applying One Health approach to Q fever ▪ Personal communications were established with principal author, detail information sourced and incorporated ▪ Moreover, this conference abstract was published in a slightly different way in 2016 as cited in reference [30]
		▪ Risk factor survey and human serology	▪ Livestock contact had strong association with Q fever	
		▪ Ruminants’ milk, vaginal swab, placenta, manure and environmental samples were tested	▪ Goat and cattle samples were positive for *C. burnetii* ▪ Birthing areas had highest concentration of *C. burnetii*	
		**Recommended**		
		▪ Health education and change in farm practices	▪ Prevent future *C. burnetii* transmission ▪ Reduce lost productivity and ensure better livelihoods	
[31] Netherlands	Review	**Recommended**		
		▪ Dispute between human health providers and veterinarians be dissolved	▪ Better Q fever control through agreed measures	▪ Communication gap between human and animal health sectors was identified in an outbreak investigation, although it was believed that both sectors were working together. One Health as a method of bridging that gap needs practical interactions rather than written words ▪ Only goat as reservoir was discussed without considering other species e.g. sheep and cattle
		▪ Better diagnostic methods	▪ Improved Q fever notifications	
		▪ Livestock vaccination	▪ Reduced human exposure through prevention of animal abortions	
[32] USA	Review	**Practiced**		
		▪ Multidisciplinary diagnostic facilities	▪ Sample testing from a range of sources	▪ Local, state and federal levels involving public and private partnerships that combine human, animal and ecological sectors helps minimize resource exhaustion in control of zoonotic diseases
		▪ Quick result production		
		▪ Less communication pitfalls among stakeholders	▪ Stewardship and collaborations	
		▪ Public-private partnerships	▪ Coordinated local responses against diseases and threats	
		▪ Joint investigation of Q fever cases		
		▪ Human and animal serology	▪ Positive Q fever serology demonstrated	
		**Recommended**		
		▪ Vector borne disease control requires human, animal and vector surveillance	▪ Shared resources and expertise ▪ Animals and humans are protected	

* HEPA: High-efficiency particulate arrestance; IFA: Immunofluorescence assay.

## Supplementary Materials

The following resource is available online at https://www.mdpi.com/1660-4601/16/5/730/s1, Table S1: Logic grids showing subject headings and keywords used for searching databases until 13 June 2018.
