# Supplementary material for: Is a One Health Approach Utilized for Q Fever Control? A Comprehensive Literature Review
Source: Int J Environ Res Public Health. 2019 Feb 28;16(5):730. doi: 10.3390/ijerph16050730 (PMC6427780; doi:10.3390/ijerph16050730)
Supplement: Supplementary file 1 [file ijerph-16-00730-s001.pdf]

Table 1. Logic grids showing subject headings and keywords used for searching databases until 13 June 2018.

**PUBMED (Logic Grid 1)**	
Q fever	One Health
“q fever”[mh] OR “q fever”[tw] OR q-fever[tiab] OR *Coxiella burnetii**[tiab] OR acute q fever[tiab] OR chronic q fever[tiab] OR query fever[tiab]	“one health”[mh] OR one health concept[tiab] OR one health initiative[tiab] OR one medicine initiative[tiab] OR “one health”[tw] OR one health*[tiab] OR “one medicine”[tw] OR one medicine*[tiab] OR “one health”[all] OR “one medicine”[all]
**EMBASE (Logic Grid 2)**	
Q fever	One Health
“q fever”/syn OR “q fever”:ti,ab OR “acute q fever”:ti,ab OR “chronic q fever”:ti,ab	“one health”/syn “one health concept”/de OR “one health initiative”/de OR “one medicine initiative”/de OR “one health”:ti,ab OR “one medicine”:ti,ab
**CINAHL (Logic Grid 3)**	
Q fever	One Health
MH q fever OR TI “q fever” OR AB “q fever” OR TI “*Coxiella burnetii*” OR AB “*Coxiella burnetii*” OR TI “acute q fever” OR AB “acute q fever” OR TI “chronic q fever” OR AB “chronic q fever” OR TI “query fever” OR AB “query fever”	TI “one health” OR AB “one health” OR TI “one medicine” OR AB “one medicine” OR MW “one health*” OR MW “one medicine*”
**SCOPUS (Logic Grid 4)**	
Q fever	One Health
“q fever” OR “q-fever” OR “acute q fever” OR “chronic q fever” OR “*C. burnetii*” OR “*Coxiella burnetii*” OR “query fever”	“one health” OR “one medicine” OR “one health*” OR “one medicine*”
**WEB OF SCIENCE (Logic Grid 5)**	
Q fever	One Health
“q fever” OR “q-fever” OR “acute q fever” OR “chronic q fever” OR “*Coxiella burnetii*” OR “query fever”	“one health” OR “one medicine” OR “one health*” OR “one medicine*”
**PsycINFO (Logic Grid 6)**	
Q fever	One Health
q fever.sh OR q fever.ti,ab OR q-fever.ti,ab OR acute q fever.ti,ab OR query fever.ti,ab OR q fever.tw OR q-fever.tw OR q fever.mp OR q-fever.mp OR *Coxiella burnetii*.ti,ab OR *Coxiella burnetii*.tw OR *Coxiella burnetii*.mp	one health.sh OR one health.ti,ab OR one health.tw OR one health.mp OR one medicine.sh OR one medicine.ti,ab OR one medicine.tw OR one medicine.mp
